# Activation of EGFR by small compounds through coupling the generation of hydrogen peroxide to stable dimerization of Cu/Zn SOD1

**DOI:** 10.1038/srep21088

**Published:** 2016-02-17

**Authors:** Vehary Sakanyan, Philippe Hulin, Rodolphe Alves de Sousa, Viviane A. O. Silva, Artur Hambardzumyan, Steven Nedellec, Christophe Tomasoni, Cédric Logé, Charles Pineau, Christos Roussakis, Fabrice Fleury, Isabelle Artaud

**Affiliations:** 1 IICiMed EA-1155, Faculté de Pharmacie, Faculté des Sciences et des Techniques, Université de Nantes, 2 rue de la Houssinière, 44322 Nantes, France; 2 ProtNeteomix, 29 rue de Provence, 44700 Orvault, France; 3 Plate-forme MicroPICell SFR Santé F. Bonamy-FED 4203/Inserm UMS016/CNRS UMS3556, 44007 Nantes, France; 4 UMR 8601, CNRS, Université Paris Descartes, PRES Paris cité, 45 rue des Saints-Pères, 75270 Paris Cedex06, France; 5 UFIP CNRS UMR 6286, Mechanism and Regulation of DNA Repair team, Faculté des Sciences et des Techniques, Université de Nantes, 2 rue de la Houssinière, 44322 Nantes, France; 6 SPC Armbiotechnology, 14, Gyurjyan str.,Yerevan 0056, Armenia; 7 Protim, Inserm U1085-Irset, Campus de Beaulieu, 35042 Rennes, France

## Abstract

Activation of cell signaling by reactive chemicals and pollutants is an important issue for human health. It has been shown that lipophilic nitro-benzoxadiazole (NBD) compounds rapidly move across the plasma membrane and enhance Epidermal Growth Factor Receptor (EGFR) tyrosine phosphorylation in cancer cells. Unlike ligand-dependent activation, the mechanism of this induction relies on the generation of hydrogen peroxide, which is involved in the activation of the catalytic site of the receptor and the inactivation of protein tyrosine phosphatase PTP-1B. Production of H_2_O_2_ during redox transformation of NBD compounds is associated with the transition of a monomeric form of Cu/Zn superoxide dismutase 1 (SOD1) to stable dimers. The highly stable and functionally active SOD1 dimer, in the absence of adequate activities in downstream reactions, promotes the disproportionate production and accumulation of intracellular hydrogen peroxide shortly after exposure to NBD compounds. The intrinsic fluorescence of small compounds was used to demonstrate their binding to SOD1. Our data indicate that H_2_O_2_ and concomitantly generated electrophilic intermediates behave as independent entities, but all contribute to the biological reactivity of NBD compounds. This study opens a promising path to identify new biomarkers of oxidative/electrophilic stress in the progression of cancer and other diseases.

Ligand-dependent activation of EGFR is an essential process governing signal transduction during cell growth, differentiation, survival, and proliferation in physiological conditions. Mutations resulting in overexpression or deregulation of the receptor impair signal transduction, leading to tumor growth^1^. Years of exposure to pollutants and chemicals can also cause an aberrant activation of EGFR-mediated signaling pathways in humans, thereby contributing to the progression of cancer^2^. Therefore, targeting EGFR remains a challenge in anti-cancer therapy and further elucidation of the complex mechanisms underlying activation of the receptor by chemically synthesized small molecules is required.

Both ligand-dependent and ligand-independent mechanisms have been shown to activate EGFR in cells. Binding of the cognate ligands EGF or TGFα to the extracellular region induces structural rearrangements favorable for dimerization of the receptor^3^,^4^. The dimerization process is a crucial step for allosteric activation of the ATP-binding site in the cytoplasmic region of EGFR and the creation of docking sites for effector proteins involved in the downstream signaling cascade^5^.

Ligand-independent phosphorylation of EGFR was initially demonstrated by studying H_2_O_2_ action on carcinoma cells overproducing the receptor^6^. It is noteworthy that the binding of EGF to the extracellular region of EGFR generates hydrogen peroxide *in vivo* and *in vitro* by an as-yet-unknown mechanism^7^,^8^. H_2_O_2_, a neutrally charged molecule, moves across the plasma membrane by simple diffusion or aquaporin-facilitated diffusion^9^. At high concentrations, H_2_O_2_ and/or related species interact with proteins, DNA and lipids and provoke cell death and tissue damage, whereas at low concentrations, they function as a second messenger in signal transduction^10^. A number of studies have demonstrated that H_2_O_2_ enhances EGFR phosphorylation in cells exposed to small chemicals^11^,^12^,^13^, small particulate matter and diesel exhaust particles^14^,^15^. However, the mechanism(s) leading to the generation of H_2_O_2_ in cells exposed to small compounds is unknown.

In addition to being affected by autophosphorylation through its intrinsic kinase activity, the phosphorylation status of EGFR is affected by protein tyrosine phosphatases, among which PTP-1B appears to be a major dephosphorylating enzyme^16^. The phosphorylation of the receptor is associated with inactivation of PTP-1B by H_2_O_2_^17^ and is a result of oxidation of a nucleophilic cysteine to unstable electrophilic sulfenic acid (Cys-SOH), which drives cyclization with a neighboring amide group and forms an inactive 3-isothiazolidinone in the catalytic site of PTP-1B^18^,^19^. Hydrogen peroxide generated during EGF binding to EGFR also oxidizes a catalytic cysteine (Cys797) to sulfenic acid in the ATP-binding pocket, which enhances auto-phosphorylation of the receptor^20^. The unstable sulfenic acid appears to be further oxidized to stable sulfinic acid (Cys-SO_2_H) with no loss of EGFR’s kinase activity^21^. These findings provide an elegant explanation for the role of hydrogen peroxide as a physiological messenger in signal transduction: by simultaneously modulating the phosphatase activity of PTP-1B and the kinase activity of EGFR through post-translational modification of a catalytic cysteine.

Nitro-benzoxadiazole (NBD), also referred to as nitro-benzofurazan, and its derivatives can interact with functional groups in proteins and have therefore been employed in developing molecular probes^22^ and fluorescent dyes for cell imaging^23^,^24^. Furthermore, NBD compounds have been used to target proteins of therapeutic significance, such as glutathione S-transferase (GSH)^25^, c-Myc oncogene^26^, and HIV integrase^27^. It has been shown that the double-ring structure of the compound 7-nitro-4-(phenylthio)benzofurazan (referred to as NBF-SPh) is subjected to redox cycling, leading to the generation of reactive oxygen species (ROS), which cause lipid peroxidation and acute death of carcinoma cells^28^. This information suggests that NBD compounds have beneficial anti-cancer effects. However, the biologically relevant properties of this important class of reactive chemicals are still obscure.

Recently, we have shown that NBD compounds bind to the extracellular region of EGFR and enhance tyrosine phosphorylation of EGFR, leading to aberrant triggering of downstream and lateral signaling in breast cancer and non-small lung cancer cells^29^. However, this enhanced phosphorylation is only partially suppressed by a neutralizing antibody and is associated with inhibition of PTP-1B activity. This finding prompted us to postulate that a ROS-mediated ligand-independent mechanism may also be involved in NBD compound modulation of receptor activity and phosphorylation.

Herein, we address the role of ROS in the activation of EGFR by NBD compounds. Our results highlight that only lipophilic NBD compounds able to move across the cell membrane can rapidly enhance EGFR tyrosine phosphorylation. The small compounds bind to and dimerize cytoplasmic Cu/Zn SOD1, and the stable dimer formed is functionally active and generates intracellular H_2_O_2_, which is responsible for the activation of the receptor in cancer cells.

## Results

### The NBD scaffold induces tyrosine phosphorylation

**Figure 1:**
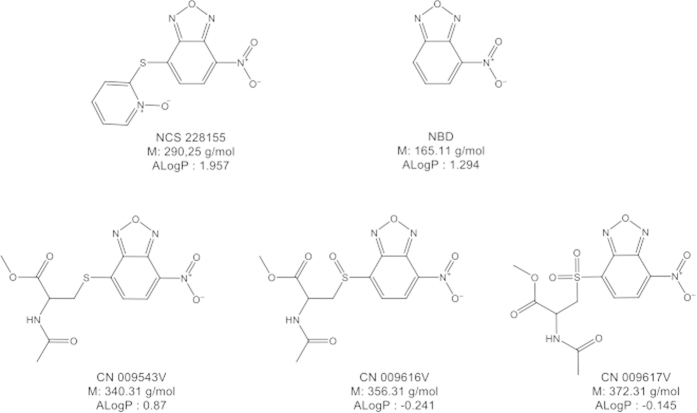
Structures and characteristics of NBD compounds. The values ALogP were calculated with Discovery Studio 4.0 software.

**Figure 2:**
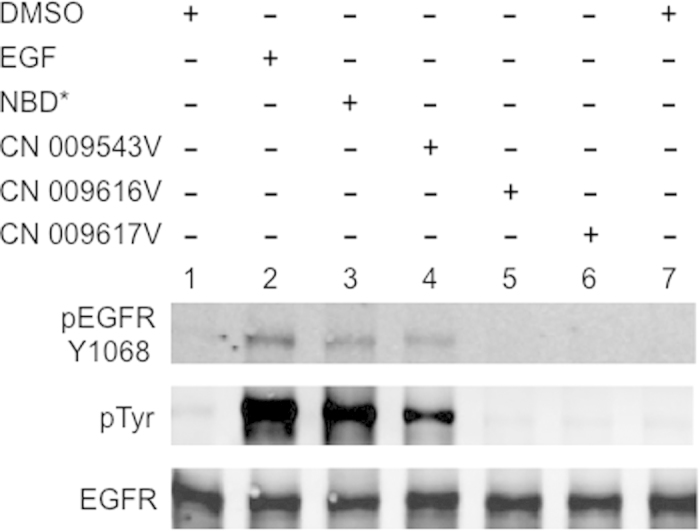
Compound NBD* and its lipophilic derivatives enhance EGFR tyrosine phosphorylation in cancer cells. MDA MB468 cells were incubated with 100 μM NBD compounds, 100 ng/ml EGF or 0.4% DMSO for 15 min.

To verify whether NBD alone could activate EGFR, we studied the action of the compound containing only the 4-nitro-2,1,3-benzoxadiazole structure (Fig. 1), herein denoted NBD*. The compound NBD* enhanced both phosphorylation at Tyr1068 of EGFR, and total protein tyrosine phosphorylation (pTyr) in MDA MB468 cells treated with the compound for 15 min (Fig. 2). These data indicate that the NBD scaffold alone is able to activate the receptor in cells.

### Only lipophilic NBD compounds move across the cell membrane and activate EGFR

Three similar compounds, CN 009543V, CN 009616V and CN 009617V, possessing, in addition to the nitro-benzoxadiazole scaffold, N-acetyl cysteine methylester or its SO or SO_2_ oxidized derivatives, respectively (see Fig. 1), were compared for their ability to activate EGFR. In contrast to CN 009543V, no tyrosine phosphorylation of the receptor was detected in cells exposed to CN 009616V or CN 009617V (see Fig. 2). This suggested that these compounds, unlike CN 009543V, could not move into the cytoplasm, and that the activation of EGFR requires the intracellular action of NBD compounds.

**Figure 3:**
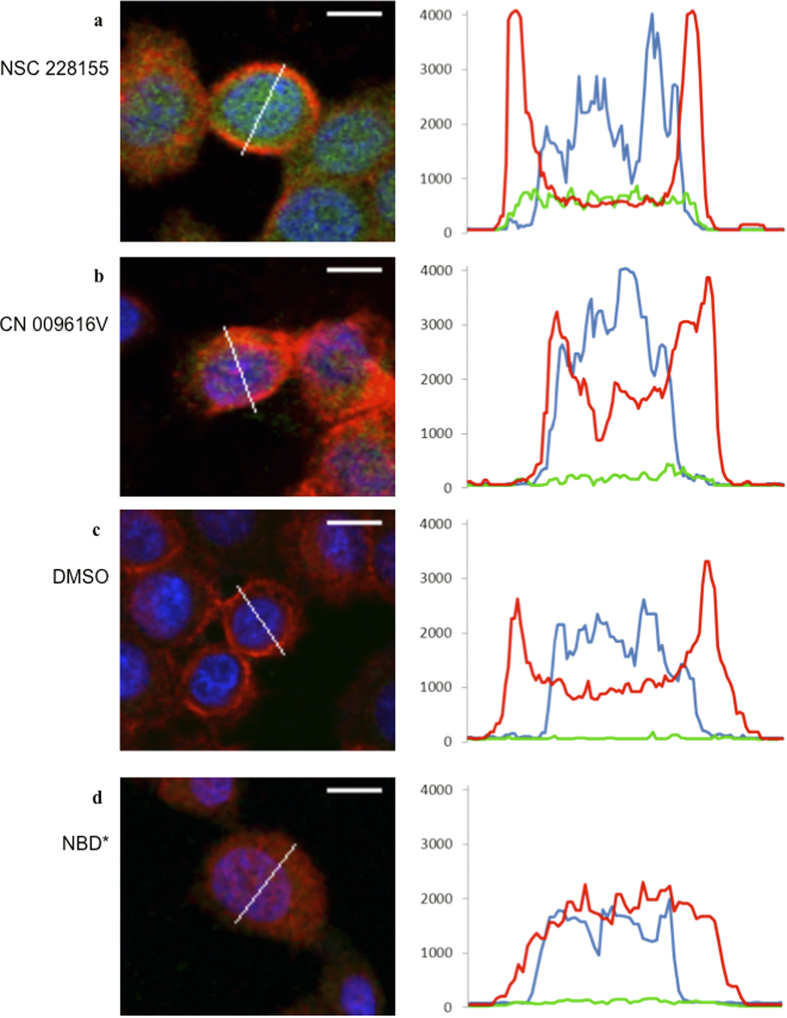
Distribution of NBD compounds in breast cancer cells detected with fluorescent microscopy. (**a**) Images of MDA MB468 cells (left) and graphical presentations of fluorescence (right) in representative cells exposed to NSC 228155 for 5 min, (**b**) CN 009616V for 15 min, (**c**) DMSO for 15 min, or (**d**) NBD* for 5 min. Plasma membrane was labelled with anti-EGFR antibody conjugated to biotin and then with Neutravidin conjugated to DyLigh 649. Wavy line shows the region scanned in representative cells; bar equals 10 μ. Fluorescence of NBD compounds (emission 520 nm) is shown in *green*, plasma membrane (emission at 650 nm) in *red*, and nucleus (420 nm) in *blue.*

We took advantage of the intrinsic fluorescence of NBD molecules (with an optimal excitation at 488 nm and emission at 520 nm under the conditions used) to determine how they are able to penetrate cells. MDA MB468 cells were incubated with NBD compounds, and images were recorded by collecting emission spectra from 420 nm to 650 nm. To assess the relative level of NBD compound distribution in cells, signal intensities specific for the compounds, and those for the plasma membrane (emission at 650 nm) and nucleus (emission at 420 nm), were plotted on the curves in different colors. The curve specific for NSC 228155 was characterized by an elevated shape (*green* color) homogeneously covering the intracellular space between two membrane peaks (*red* color), including the nuclear space (*blue* color) (Fig. 3a and Fig. S1a). In contrast, weak fluorescence was detected at 520 nm in cells exposed to CN 009616V for 15 min (Fig. 3b and Fig. S1b). The level of the signal for this compound was only slightly higher in whole cells compared with the background fluorescence recorded in the control assay with cells treated with vehicle (Fig. 3c and Fig. S1c).

Although the intracellular distribution of NBD* could not be detected in cells because of its low emission wavelength, two polar peaks of the plasma membrane disappeared in the majority of cells after exposure (Fig. 3d and Fig. S1d). This suggests that the NBD* compound, at the concentration used, drastically disturbs the plasma membrane of cells.

Thus, NBD compounds exhibit different abilities to penetrate cells. Compounds NSC 228155 and CN 009543V (see also below) rapidly move across cell membranes and disperse within both cytoplasmic and nuclear compartments, whereas compounds CN 009616V and CN 009617V cannot move across the lipid bilayer. Such behavior of NBD compounds is consistent with the lipophilicity of small molecules, according to calculated log P values (see Fig. 1). Compounds NSC 228155 and CN 009543V have a positive log P and could enter cells and enhance EGFR tyrosine phosphorylation. In contrast, CN 009616V and CN 009617V have a negative log P and could not move across the plasma membrane or damage the lipid bilayer to penetrate cells. These latter two compounds therefore do not induce EGFR tyrosine phosphorylation in cells, at least under the conditions used.

### NBD compounds activate EGFR through redox transformation

**Figure 4:**
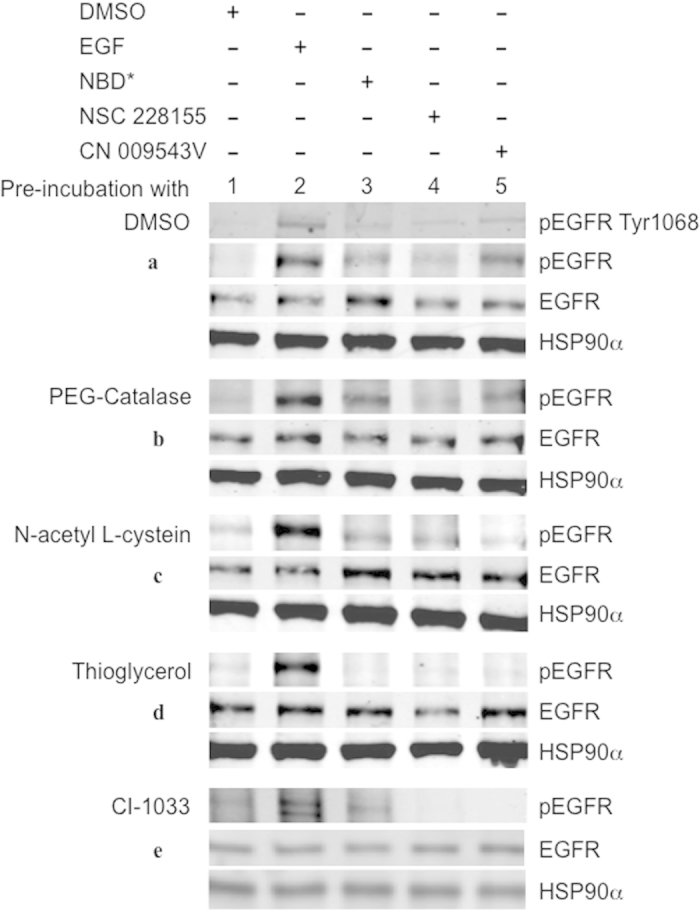
Profile of EGFR tyrosine phosphorylation in cells pre-incubated with antioxidants or EGFR tyrosine kinase inhibitor and exposed to NBD compounds. MDA MB468 cells were pre-incubated with 0,2% DMSO (**a**) 500 U/ml PEG-catalase (**b**), 5 mM NAC (**c**), 5 mM thioglycerol (**d**) or 2 μM CI-1033 (**e**) and exposed to NBD compounds (100 μM), EGF (100 ng/ml) or vehicle for 15 min.

Different types of antioxidants were studied for their ability to prevent tyrosine phosphorylation of EGFR in MDA MB468 cells exposed to NBD compounds (Fig. 4). Pre-incubation of cells with PEG-catalase weakly suppressed tyrosine phosphorylation of the receptor in cells exposed to NBD compounds (Fig. 4b), whereas N-acetyl-L-cysteine (NAC) suppressed protein phosphorylation relatively strongly (Fig. 4c). In contrast, thioglycerol completely abolished protein tyrosine phosphorylation in cells exposed to any of the NBD compounds (Fig. 4d). Moreover, all three antioxidants very weakly affected tyrosine phosphorylation of EGFR in cells exposed to EGF in parallel assays.

The prevention of EGFR tyrosine phosphorylation by thioglycerol and, to a lesser extent by NAC, in cells exposed to NBD compounds indicated that these compounds generate ROS within cells and that this is an early event preceding the activation of the receptor. Lipophilic NBD compounds, once transferred into the cell, apparently participate in redox cycling by generating H_2_O_2_ required for activation of EGFR.

### NBD compounds rapidly generate hydrogen peroxide within cells

**Figure 5:**
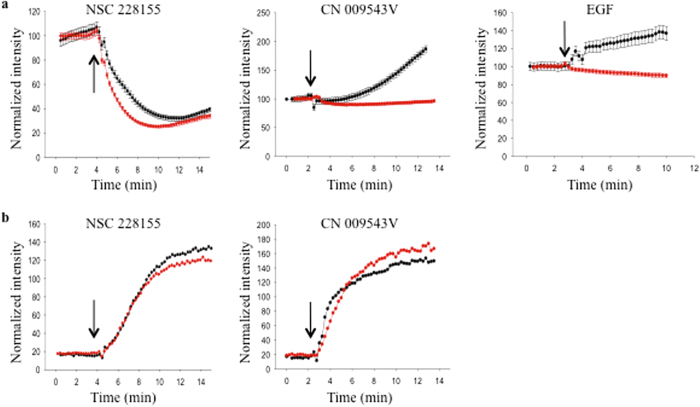
Detection of ROS in living cells exposed to NBD compounds by fluorescent microscopy. MDA MB468 cells were incubated with or without PEG-catalase (500 U/ml) at 37 °C for 20 min. CellROX Deep Red reagent was added and incubation continued for 30 min to reach the steady state (controlled by fluorescence) followed by addition of NBD compounds (100 μM) or EGF (500 ng/ml). Fluorescence was simultaneously monitored at 665 nm to detect ROS (**a**) and at 520 nm to detect NBD compounds (**b**). Fluorescent curves for cells pre-incubated without PEG-catalase are shown by black circles and for cells pre-incubated with PEG-catalase by red circles. The arrow indicates addition of NBD compounds or EGF to cultures. Data are presented as means ± SEM.

To assess the production of intracellular ROS in response to exposure to NBD compounds in real time, a sensitive method to detect oxidized fluorescent reagent (emission at 665 nm) in living cells was used. MDA MB468 cells were incubated with CellROX Deep Red to establish the fluorescence baseline, and then NSC 228155 or CN 009543V was added to the culture. Fluorescence at 520 nm and 665 nm was monitored by video-camera to simultaneously identify the intracellular production of ROS and the penetration of NBD compounds into cells. The fluorescence intensities of 20–30 cells were plotted for each assay to evaluate the relative amount of intracellular ROS generated during exposure to NBD compounds (Fig. 5).

A rapid increase during the first 5 min, followed by stabilization of 520 nm fluorescence curves, characterized both NSC 228155 and CN 009543V penetration, indicating that the concentration of these compounds rapidly reached saturation level within cells (Fig. 5b). However, the shapes of the fluorescence curves recorded at 665 nm were different for NSC 228155 and CN 009543V, suggesting variations in the kinetics of their redox-cycling transformations. The curve of the NSC 228155-mediated response was biphasic, characterized by a decrease in the fluorescence signal during the first 5 min, followed by a slow increase that remained below the baseline (Fig. 5a). The curve of the CN 009543V-mediated response gradually rose after addition of the compound up until the end of exposure to the compound.

In an effort to identify intracellular H_2_O_2_, fluorescence responses were also monitored after pre-incubating cells with PEG-catalase. The increase in the fluorescence curve at 665 nm was moderately suppressed in the cells exposed to NSC 228155 and fluorescence was completely suppressed after exposure to CN 009543V (see Fig. 5a), indicating that H_2_O_2_ is involved in redox cycling.

To confirm this assumption, ROS production was evaluated within cells after exposure to EGF, which is known to generate H_2_O_2_ during binding to the receptor^7^. Exposure to this cognate ligand clearly led to a gradual rise of a 665-nm fluorescence curve, whereas pre-incubation of cells with PEG-catalase significantly decreased the fluorescence signal in EGF-induced cells (see Fig. 5a). This shows that exogenously added catalase suppresses the production of intracellular H_2_O_2_ during binding of the cognate ligand to its receptor, in agreement with various studies^7^,^8^,^20^.

Thus, modulations of the fluorescence intensity at 665 nm detected within cells during exposure to NBD compounds support the generation of hydrogen peroxide. Compound NSC 228155 exhibited scavenging activity during the first 5 min, followed by intracellular accumulation of H_2_O_2_, as confirmed by attenuation of the ROS-specific fluorescence signal when cells are pre-incubated with PEG-catalase (see Fig. 5a). In corroboration with this result, enhanced EGFR tyrosine phosphorylation was detectable with a 5 min delay after exposure to NSC 228155^29^. Compound CN 009543V resulted in the generation of intracellular H_2_O_2_ almost as soon as the cells were exposed to the compound (see Fig. 5a). The difference in kinetics of H_2_O_2_ generation by compounds NSC 228155 and CN 009543V was apparently related to the action of different moieties connected to the NBD scaffold (see Fig. 1).

Thus, *in cellulo* assays revealed in real-time that lipophilic NBD compounds rapidly undergo redox cycling and generate H_2_O_2_, which, if it reaches a critical level within cells, could activate EGFR.

### NBD compounds induce dimerization of SOD1 in cells

Generation of intracellular ROS by NBD compounds may be related to the enzymatic action of superoxide dismutase, catalase and/or glutathione peroxidase, which are responsible for the protection of human cells against toxic redox cycling^30^. Given that these enzymes are functionally active in dimeric or tetrameric forms in animal cells, we assessed the oligomerization state of these proteins after treatment of cancer cells with NBD compounds.

Surprisingly, a significant proportion of Cu/Zn SOD1, known to be mainly localized in the cytosol^31^, was present as a stable 32-kDa dimer in the soluble fraction of extracts of MDA MB 468 breast cancer cells, as well as in DU145 prostate cancer cells treated with NSC 228155, but not with EGF, for 5 min or 10 min (Fig. S2a). No changes were detected in the oligomerization and expression of catalase and glutathione peroxidase after a short exposure to NSC 228155 (Fig. S2b and S2c).

**Figure 6:**
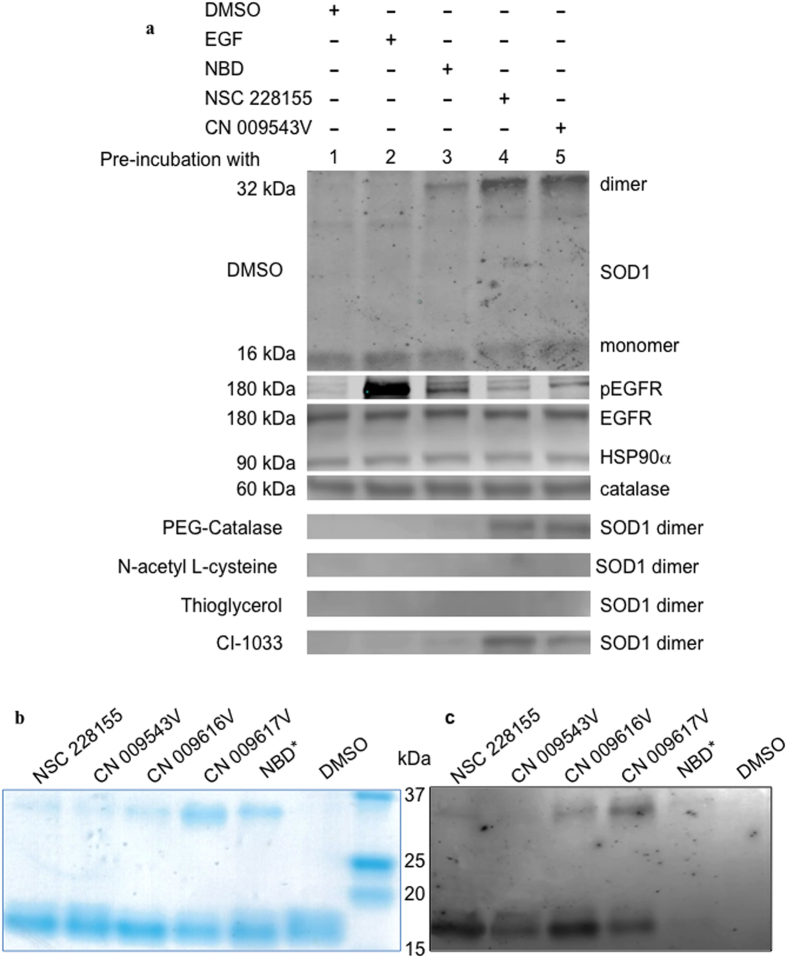
Dimerization of SOD1 in cancer cells exposed to lipophilic NBD compounds, and SOD1 exposed to NBD compounds *in vitro*. (**a**) Western blot analysis of proteins in MDA MB468 cells pre-incubated with NAC (5 mM), PEG-catalase (500 U/ml), thioglycerol (5 mM) or CI-1033 (2 μM) for 30 min, and then exposed to NBD compounds (100 μM) or EGF (500 ng/ml) for 15 min. SOD1 monomers were present in all cells (shown only in cells pre-incubated with DMSO). (**b**) Detection of NBD compounds bound to purified human SOD1 with Coomassie staining. (**c)** Fluorescence detection of NBD compound/SOD1 complexes blotted onto NC membrane using a Typhoon 9410 scanner; the excitation wavelength was 488 nm and the emission wavelength was 526 nm.

Further analysis showed that lipophilic compounds NSC 228155 and CN 009543V provided higher yields of stable SOD1 dimers than NBD* at equimolar concentrations in MDA MB468 cells (Fig. 6a). Meanwhile, no dimeric forms of SOD1 were detected in cells treated with EGF (500 ng/ml), suggesting specific action of NBD compounds on SOD1. Indeed, pre-incubation of cells with the irreversible tyrosine kinase inhibitor CI-1033, which binds to Cys797 of EGFR^32^, attenuated tyrosine phosphorylation of the receptor (Fig. 4e) but did not affect SOD1 dimerization in cells exposed to NBD compounds (see Fig. 6a). Furthermore, thioglycerol and NAC, completely abolished SOD1 dimerization in cells exposed to NSC 228155, CN 009543V or NBD* (see Fig. 6A), indicating that electrophilic species generated during NBD redox cycling are associated with the activity of SOD1 in the cytoplasm.

### EGFR phosphorylation depends on the dimerization state of SOD1 in cells exposed to NBD compounds

The dimerization state of wild type and dimerization-impaired mutant forms determines both the stability and catalytic activity of Cu/Zn SOD1 in eukaryotic cells^33^,^34^. The SOD1 homodimer is a stable structure in cells; however, it is disrupted during electrophoresis under reducing conditions in a denaturing gel. The SOD1 dimer induced by NBD compounds is not disrupted during electrophoresis. This property was used to further investigate the relationship of SOD1 dimerization with EGFR phosphorylation in cells.

**Figure 7:**
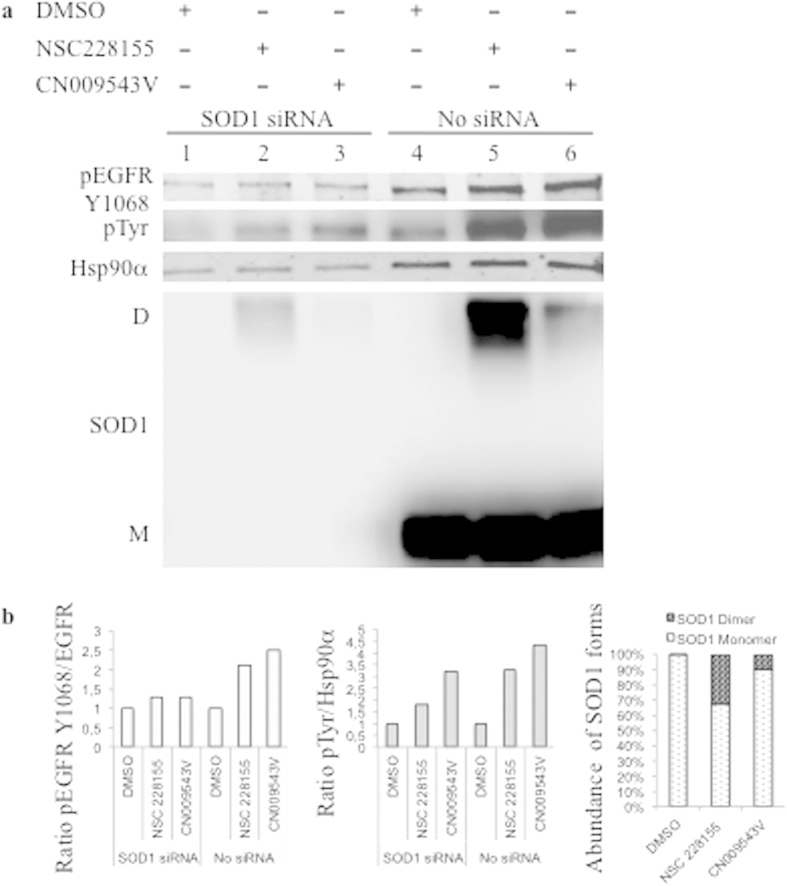
Effects of SOD1 siRNA interference on tyrosine phosphorylation of EGFR, and expression of SOD1 in cancer cells exposed to NBD compounds. (**a**) MDA MB468 cells were transfected with SOD1 siRNA (SOD1 siRNA) or not transfected (No siRNA), and exposed to 0.4% DMSO or 100 μM NSC 228155 or CN 009543V for 10 min. Cells extracts were analyzed by western blotting. Shown are results from a representative experiment. (**b**) The signal intensity of protein bands was quantified with C-Digit software (Li-COR). The relative rate of EGFR phosphorylation was normalized to the loading control Hsp90α, and the value obtained was compared with that of cells exposed to a vehicle (equal to 1 on the histograms). The relative abundance of monomeric and dimeric forms of SOD1 was quantified as the ratio of corresponding bands (%) in non-transfected cells.

**Figure 8:**
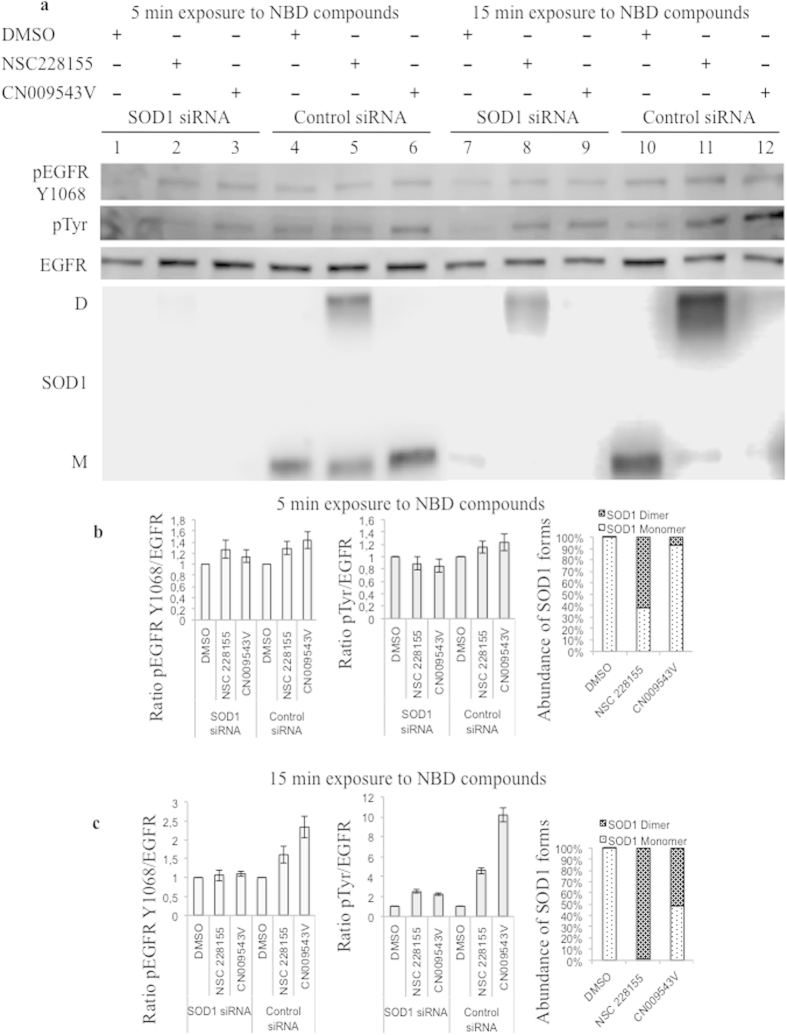
Comparison of EGFR tyrosine phosphorylation and SOD1 dimerization in cells transfected with SOD1 siRNA and scrambled siRNA after exposure to NBD compounds. (**a**) Western blot analysis of EGFR phosphorylation and SOD1 dimerization after 5 min and 15 min exposures to 100 μM NBD compounds in MDA MB468 cells transfected with SOD1 siRNA or scrambled siRNA (Control siRNA). Quantitative analysis of the kinetics of EGFR phosphorylation and SOD1 dimerization after 5 min (**b**) and 15 min (**c**) exposures to 100 μM NBD compounds. The relative rate of EGFR phosphorylation was normalized to non-phosphorylated EGFR. The relative abundance of monomeric and dimeric forms of SOD1 was quantified as the ratio of corresponding bands (%) in the cells transfected with scrambled siRNA.

To examine whether activation of EGFR by NBD compounds depends on the SOD1 dimerization state, tyrosine phosphorylation of the receptor was assessed in RNA interference experiments. Because human SOD1 is a long-lived protein^35^, longer cultivation of cells transfected with siRNA was required to increase the silencing effect of a SOD1-specific siRNA in MDA MB466 cells. It was shown that after SOD1 siRNA transfection for a total of 90 h, the amount of SOD1, especially monomeric protein, was remarkably decreased in cells exposed to a vehicle or the compounds NSC 228155 or CN 009543V for 10 min, compared with that in non-transfected cells in parallel assays (Fig. 7). SOD1 expression was also decreased in cells transfected with scrambled siRNA (negative control), but it was much higher than that in the cells transfected with SOD1-specific siRNA, proving the efficacy of the RNA interference (Fig. 8a).

Notably, tyrosine phosphorylation of EGFR, and not its expression, was clearly attenuated in SOD1 siRNA-treated cells after exposure to NBD compounds for 5 and 15 minutes, compared with that in cells transfected with a negative control, scrambled siRNA (see Figs 7 and 8). These results confirmed that EGFR tyrosine phosphorylation is enhanced through the action of SOD1 shortly after exposure of cells to NSC 228155 or CN 009543V.

Next, the enhanced EGFR tyrosine phosphorylation was shown to be associated with an augmentation of the proportion of SOD1 dimer formed in cells transfected with scrambled siRNA, as a function of the duration of exposure to NBD compounds (see Fig. 8a). Although EGFR phosphorylation was not yet significant after a 5 min exposure, it was clearly enhanced after a 15 min exposure to NBD compounds, with an increase in the amount of SOD1 dimers. Quantitative assessment demonstrated that the abundance of SOD1 dimers, induced by NSC 228155 and CN 009543V after a 5 min exposure, constituted approximately 62% and 7% (Fig. 8b), respectively, and after a 15-min exposure the amount of SOD1 dimers reached approximately 98% and 53% in the cells transfected with scrambled siRNA (Fig. 8c). Thus, EGFR tyrosine phosphorylation is dependent on the transition of a monomeric form of SOD1 to dimeric forms in MDA MB468 cells shortly after exposure to NSC 228155 and CN 009543V. Hence, these compounds induce the formation of functionally active dimeric forms of SOD1, and this is supported by the gradual increase in intracellular H_2_O_2_ after exposure to NSC 228155 and CN 009543V in real-time assays (see Fig. 5). The progression of the proportion of SOD1 dimers was still apparent in SOD1 siRNA-treated cells (see Fig. 8a), suggesting that residual dimers are related to incomplete knockdown of SOD1 in MDA MB468 cells treated with NBD compounds.

Together, our results show that lipophilic NBD compounds primarily induce the formation of a stable SOD1 dimer that is functionally active and, in the absence of adequate catalase and glutathione peroxidase activities, leads to the accumulation of intracellular H_2_O_2_, which in turn activates EGFR. The formation of SOD1 dimers, which tolerate the action of SDS and β-mercaptoethanol during electrophoresis, supports a strong, apparently irreversible interaction between protein monomers, with participation from the reduced NBD compounds in cells.

### NBD compounds bind to SOD1

To examine whether NBD compounds bind to SOD1, the purified protein and NBD compounds (at molar ratio of 8:1) were incubated in non-reducing conditions (without β-mercaptoethanol), and the reaction products were separated by SDS-PAGE in the presence of β-mercaptoethanol. All compounds promoted dimerization of SOD1 with different levels of efficiency, as judged by staining of protein bands (Fig. 6b). However, no fluorescence was detected from protein bands in a gel by scanning at different wavelengths with the Typhoon imaging system, probably because the polyacrylamide gel extinguished the fluorescence. To increase the signal intensity, the separated reaction products were blotted from the gel onto a nitrocellulose membrane and then scanned with the Typhoon. Different intensities of fluorescence were detected for monomeric and dimeric protein bands at 526 nm, demonstrating that NBD compounds bind to both forms of SOD1 (Fig. 6c). For dimers, the highest fluorescence intensity was recorded for the CN 009617V/SOD1 complex, and the weakest was recorded for the CN 009543V/SOD1 complex.

Thus, fluorescence emitted from protein bands indicates that electrophilic intermediates of NBD compounds bind to SOD1 monomers, resulting probably in covalently cross-linked protein subunits, which are highly stable *in vivo* and *in vitro*.

To gain insight into NBD compound interactions, docking analysis was performed with a high-resolution SOD1 homodimer structure, 4A7U^36^. Lipophilic and non-lipophilic NBD compounds were accommodated within the hydrophobic cavity constituted by Val7 Val158 in the dimerization region or interacted with other putative sites involved in protein dimerization (Figs S3, S4 and Table S1). However, docking data for SOD1 dimer stabilizers^37^ has not been confirmed in recently obtained co-crystal structures of some stabilizers bound to the protein^36^. Therefore, mechanistic experimentation is required to elucidate the binding mode of NBD compounds to SOD1.

### Toxicity of NBD compounds

Accumulation of intracellular H_2_O_2_ after exposure to NBD compounds may have harmful effects on cells. The viability of MDA MB468 breast cancer cells and NCTC 2544 transformed keratinocytes was determined after incubation with NBD compounds for 48 h. Lipophilic compounds NBD*, NCS 228155 and CN 009543V were more toxic to cancer cells than to transformed keratinocytes (Table S2). The toxicity of NCS 228155 was more pronounced than that of NBD* or CN 009543V in these cell lines. Compounds CN 009616V and CN 009617V were clearly non-toxic within the range of concentrations tested, which could be related to their inability to enter cells and target vital functions.

Notably, NSC 228155 and CN 009543V (and surprisingly, EGF at 500 ng/ml) induced morphological changes in MDA MB468 cells by forming blebbed structures on the surface of many cells, which were well visible by fluorescence microscopy (Fig. S5). It is known that protrusion of the nuclear or other cellular surfaces results in blebbing, which appears to be associated with detachment of the membrane and cell death^38^. Thus, the excess H_2_O_2_ generated by stable SOD1 dimers may cause membrane damage and further death of cells exposed to NBD compounds, although other mechanisms should not be excluded.

## Discussion

We have recently shown that small NBD compounds enhance EGFR phosphorylation in the absence of a cognate ligand and thereby trigger aberrant signaling in cancer cells^29^. Our new findings highlight that the initial event in the activation of EGFR is related to the generation of intracellular H_2_O_2_ in cells exposed to NBD compounds. This is also important for understanding the biological reactivity of NBD and similar compounds in cells.

We show that the compound NBD* alone enhances tyrosine phosphorylation of EGFR, demonstrating that the 4-nitro-2,1,3-benzoxadiazole scaffold is responsible for aberrant biological consequences in cells. Pre-incubation of cells with thioglycerol suppressed tyrosine phosphorylation of the receptor after exposure to NBD* or its lipophilic derivatives NSC 228155 or CN 009543V. This preventive effect of thioglycerol indicates that (i) NBD compounds become active within cells if molecular oxygen is available to initiate redox transformations and (ii) the generation of ROS precedes the activation of EGFR. Moreover, RNA interference experiments and real-time fluorescence detection of intracellular ROS revealed that H_2_O_2_ is the species responsible for the activation of EGFR in cells. Notably, highly stable dimeric forms of SOD1 bound to electrophilic NBD compounds primarily produce the H_2_O_2_.

Our results are in agreement with the reduction pathway experimentally established for a patented compound, NBF-SPh, which is considered to be a potent generator of superoxide and hydrogen peroxide^28^. This compound participates in redox cycling by reduction of the 7-nitro group, leading to the generation of O_2_^−^ and H_2_O_2,_ and formation of concomitant electrophilic intermediates (*ibid*). According to our data, the generation of H_2_O_2_ is markedly strengthened by rapid dimerization of SOD1 in the cytoplasm after exposure of cells to NBD compounds, and the induced stable SOD1 dimer plays a crucial role in the intracellular production of H_2_O_2_. The involvement of SOD1 in the electrochemical reduction of NBD compounds appears to maintain the balance between O_2_^−^ (spontaneously generated) and reactive and toxic hydrogen peroxide (enzymatically produced) in cells. However, in the absence of adequate downstream reactions, stable and active dimers of SOD1 result in the accumulation of H_2_O_2_ within cells. This discovery is missing and important element in understanding how NBD compounds enhance tyrosine phosphorylation of EGFR, and trigger the receptor’s activity in cells.

We propose a plausible explanation of the mechanism of ligand-independent activation of EGFR, which relies on the action of H_2_O_2_ generated by the stable SOD1 dimer, which is induced by lipophilic NBD compounds. We have previously demonstrated that the enhanced tyrosine phosphorylation of EGFR by compounds NSC 228155 or CN 009543V is associated with inhibition of PTP-1B activity in MDA MB468 cancer cells^29^. Oxidation of a catalytic cysteine to sulfenic acid in PTP-1B by hydrogen peroxide has been shown to inactivate the enzyme^18^,^19^, and this results in enhancing tyrosine phosphorylation of the receptor^17^. Moreover, hydrogen peroxide, locally generated during the binding of a cognate ligand to the extracellular region of EGFR or added to the cell culture, promotes the sulfenylation of Cys797 required for the activation of the receptor^20^. Attenuation of the activating effect of electrophilic NBD compounds on EGFR tyrosine phosphorylation while Cys797 is covalently linked to the inhibitor CI-1033 (see Fig. 4e), supports the protection of the catalytic cysteine from H_2_O_2_ in the exposed cells. Thus, when H_2_O_2_ produced after exposure to lipophilic NBD compounds was to reach a critical intracellular concentration, it would inhibit the catalytic site in PTP-1B and activate the catalytic site in EGFR and thereby, trigger downstream signaling in cells.

Another important finding of our results is related to the formation of a covalent SOD1 dimer as a result of the binding of an electrophilic NBD intermediate to the protein. An aberrant covalent heptasulfane bridge (Cys-S_5_-Cys) has been identified in the SOD1 homodimer *in vitro* and *in vivo* between cysteine residues^39^. These residues are normally involved in a reversible disulfide linkage between monomers to form a structurally stable and functionally active enzyme^40^. Mutant SOD1 proteins with impaired dimerization usually exhibit a high tendency to aggregate into larger structures, contributing to the pathophysiology of familial amyotrophic lateral sclerosis, ALS (www.alsod.iop.kcl.ac.uk). Different strategies have been proposed to inhibit the aggregation of mutant SOD1 into the larger oligomeric structures known to be the most common cause of ALS^41^.

In light of these observations, our data show that the formation of stable SOD1 dimers is possible through the NBD compound-induced linkage of monomers in cells. We took advantage of the intrinsic fluorescence of NBD compounds to detect bound compound-protein complexes *in vitro*, by a blotting technique without antibodies. This approach is simple and can be used to visually distinguish structural forms of the protein bound to a small molecule, thus providing a great advantage over other detection methods for molecular interactions.

Regarding the binding mode of NBD compounds, an NBD-hexanol derivative covalently binds to glutathione in the resolved 3D compound-bound structure of the glutathione *S*-transferase enzyme^42^. We demonstrated that compounds CN 009543V, CN 009616V and CN 009617V, which differ in the sulfur oxidation state, most likely covalently bind to SOD1 *in vitro* (see Fig. 6c). Hence, reducing NBD compounds is possible regardless of their lipophilicity outside of cells if conditions are appropriate, such as in a slightly alkaline medium, to promote the formation of reactive electrophilic species and binding to protein targets. In contrast to NBD-thioether CN009543V, NBD-sulfoxide CN009616V and NBD-sulfone CN009617V appeared to stabilize the electrophilic species formed, explaining their higher reactivity *in vitro* towards nucleophilic amino acids in SOD1 monomers. This knowledge might have particular value for investigation of ALS and possibly other neurodegenerative diseases.

Several lines of data described previously have suggested that the binding of NBD compounds to the extracellular region of EGFR is involved in enhancing tyrosine phosphorylation of the receptor^29^. However, this study definitively indicates that the primary event is the generation of H_2_O_2_, which activates EGFR in cancer cells. Nevertheless, this does not exclude the contribution of electrophilic NBD intermediates binding to EGFR to modulate tyrosine kinase activity. Given that CN 009616V and CN 009617V could not enter cells and these compounds were inactive towards EGFR in exposed cells, dimerization of the receptor appears to be an independent event. Dimerization may contribute to enhancing the protein’s phosphorylation, for instance, through stabilization of the compound-protein complex, which is favorable for keeping the receptor active for a longer time. Further investigations are required to understand how NBD compounds enhance tyrosine phosphorylation by binding to the extracellular region of the receptor on the cell surface.

NBD compounds give rise to two types of reactive molecules, ROS and electrophilic NBD intermediates, which are integrally associated with redox cycling. Once generated, these molecules behave as independent entities and are potentially able to rapidly attack large numbers of target proteins involved in the maintenance of intracellular proteostasis. This view of reactive molecules with a dual mechanism of action is important for better understanding of the biological consequences of atmospheric pollutants containing compounds that undergo redox transformations in cells. Unlike ROS, a role for electrophilic compounds in cell signaling and other cellular processes is only now emerging. In this context, studying interactions of fluorescent NBD compounds with proteins is a promising way to elucidate the overall response of the human proteome to oxidative/electrophilic stress caused by reactive molecules. This could be a prerequisite to identify new diagnostic and therapeutic biomarkers of cancer and other diseases.

## Materials and Methods

### Chemicals and reagents

Compound NSC 228155 was obtained from the Drug Synthesis and Chemistry Branch of NCI (http://dtp.nci.nih.gov). 4-nitro-2,1,3-benzoxadiazole (referred to as NBD* in this study) as well as SOD1 siRNA (duplex), scramble siRNA (negative control), FITC-conjugated siRNA and siRNA transfection reagent were purchased from Santa Cruz Biotechnology. Catalase, PEG-catalase, thioglycerol, NAC, and compound CI-1033 were from Sigma-Aldrich. Stock solutions of chemical compounds in DMSO were stored at −80 °C and diluted to final concentration in serum-free culture medium just before respective assays. Primary anti-EGFR antibody (epitope in the cytoplasmic region), and secondary antibodies conjugated to DyLight-680 or DyLight-800, and Neutravidin conjugated to fluorescent dyes DyLight 649 or DyLight-800, as well as protease and phosphatase inhibitor cocktail were from Thermo Scientific. Mouse anti-pTyr (pTyr-100) mAb conjugated to biotin and rabbit anti-catalase antibodies were from Cell Signaling Technologies. Human protein EGF, goat anti-human EGFR polyclonal antibody conjugated to biotin, mouse anti-human pEGFR Tyr1068 and anti-human Cu/Zn SOD1 mAbs, and rabbit anti-Hsp90α polyclonal antibody (loading control of cytoplasmic proteins) were from R&D Systems. Recombinant human protein SOD1 was purchased from AcroBiosystems. CellROX Deep Red Reagent, Prolong Gold antifade mountant and paraformaldehyde (PFA) were from Life Technologies. Tissue culture treated 8-well μ-Slides were from Ibidi. Goat anti-mouse IgG conjugated to Horseradish Peroxidase and WesternSure chemiluminescent substrate were from Li-Cor.

### Synthesis of NBD compounds

The synthetic route for NBD compounds is depicted in Fig. S6. Synthesis of compounds CN 009543V and CN 009616V was described previously^43^. Compound CN 009617V was newly synthesized and characterized in this study. Experimental procedures as well as spectroscopic data of NBD compounds are detailed in Supplementary Text. Excitation at 488 nm and emission at 520–540 nm were found optimal to detect binding of NBD compounds in the supernatant fraction of cell lysates (Fig. S7) and therefore these wavelengths were used in biological assays in this study.

### Cell culture

Breast cancer cell line MDA MB468, overproducing EGFR^44^, was grown in Dulbecco modified eagle medium (DMEM) supplemented with 10% serum in a humidified atmosphere of 5% CO_2_ at 37 °C. At 70–80% confluence, cells were twice washed with PBS (pH 7.4) and harvested by trypsin treatment and transferred at a density of ∼6 × 10^6^ cells/ml into 6-well plates for 2 h. The medium was eliminated and cells, after washing with PBS, were deprived of serum for 18 h. Cells were washed with PBS and suspended in serum-free DMEM containing NBD compounds (100 μM) or EGF (100 ng/ml or 500 ng/ml) or DMSO as vehicle (0.4%) at 37 °C for 15 min. To assess the action of PEG-catalase (500 U/ml), mono-thioglycerol (5 mM), NAC (5 mM) or CI-1033 (2 μM) cells were pre-incubated with these agents for 30 min and treated as described above.

### Protein extraction

Cells were washed twice with cold Tris-buffered saline (TBS) and lysed with Pierce® IP lysis buffer containing 1:100 diluted protease and phosphatase inhibitor cocktail at 4 °C. The cell lysates were harvested and centrifuged at 13.000 g for 15 min to collect supernatant fractions for assays. Total protein concentration was determined using bovine serum albumin as standard.

### Western blot detection of proteins

Equal amounts of protein samples were separated by electrophoresis on 4–15% gradient gels in SDS-Tris-Glycine buffer and blotted onto nitrocellulose membrane with a Trans-Blot Turbo system (Bio-Rad). Membranes were blocked in a solution of 5% BSA/TBS containing 0.1% Tween. Fluorescent detection was performed at 700 nm and 800 nm with an Odyssey infrared imagery system (Li-COR) and protein bands were analyzed as described previously^45^. Chemiluminescence detection was done with C-Digit scanner (Li-COR). The blotted membranes were also probed with anti-HSP90α or anti-α-tubulin antibodies to assess the amount of loaded proteins.

### Immunofluorescence microscopy

MDA MB468 cells (5 × 10^4^ cells per well) were transferred into 8-well μ-Slides (Ibidi) and serum-starved for 18 h then exposed to NBD compounds (100 μM) for 5 min or 15 min at 37 °C. The cells were washed twice with cold PBS, and immediately treated with 4% paraformaldehyde at room temperature for 15 min. To visualize the plasma membrane, cells were incubated with 250-fold diluted biotinylated anti-EGFR antibody for 60 min at room temperature, washed with PBS, and then incubated with Neutravidin-DyLight 649 (dilution 1:1000) at 37 °C for 60 min and washed with PBS. To visualize the nucleus, cells were stained with Hoechst 33342 (dilution 1:1000) for 15 min at room temperature. The slide surface with immobilized cells was covered with Prolong Gold antifade reagent at room temperature in the dark for 48 h. The cells were viewed with a laser-scanning confocal microscope (Nikon A1RSi) and a wide-field microscope (Nikon Eclipse E800). The images were recorded with NIS Element software (V4.2) and processed with ImageJ software (NIH v1.49).

### Measurement of intracellular ROS

Production of ROS was assessed in living cells by recording images using a widefield fluorescent microscope (Leica DMI6000B) equipped with a video camera (Coolsnap HQ2). Commonly known ROS-specific probes have an emission wavelength of 520 nm, close to that emitted by NBD compounds. Thus, to exclude an intrinsic fluorescence of NBD compounds, the CellROX Deep Red Reagent was chosen, which readily diffuses into cells and is detected at emission 665 nm when oxidized by ROS (www.lifetechnologies.com). Starved MDA MB468 cells were incubated in 8-well μ-Slides overnight and then placed in a 5% CO_2_ thermo-stated living chamber at 37 °C during image acquisitions. CellROX Deep Red reagent (5 μM final concentration) was mixed with DMEM and added to each well 30 min before image recording. NBD compounds and EGF prepared in DMEM were added to respective wells while continuing image recording. Fluorescence of NBD compounds and CellROX dye were simultaneously recorded at 15-sec intervals at 520 nm and 700 nm, respectively. CellROX signal intensity was taken as a baseline in each assay for 5 minutes before NBD compounds or EGF were added. Each image within the acquisition field of 20–30 cells was individually processed.

### RNA interference knockdown of SOD1

MDA MB468 cells were seeded (1.5 × 10^5^ cells/well) in a six well tissue culture plate and incubated in DMEM with serum for 18 h. The optimal concentration of siRNA to be transfected was monitored with fluorescence microscopy using control FITC-conjugated siRNA. Transfection of siRNAs was performed with 60 pmol of human SOD1 siRNA (a pool of 3 target-specific 19–25 nt siRNAs designed to knockdown gene expression) or scrambled siRNA (negative control) for 6 h according to manufacturer’s recommendations (Santa Cruz Biotechnology). Transfected cells were cultured in DMEM supplemented with serum for 18 h and then in serum-free medium for 72 h before exposure to NBD compounds as described above.

### *In vitro* binding of small molecules to proteins

NBD compound binding reactions were carried out with purified SOD1 at 18 °C for 30 min at the initial pH of 7.4, and reaction mixtures were run on two gels. One gel was visualized with Coomassie stain. Another gel was blotted onto NC membrane and scanned with a Typhoon 9410 imaging system (GE Healthcare) to detect intrinsic fluorescence of small molecules (at the emission wavelength of 526 nm) bound to SOD1. The Typhoon system is equipped with multiple filters, enabling fluorescence to be detected over a wide range of wavelengths.

### Molecular docking

*In silico* modeling of small compound interactions with SOD1 was performed with the AutoDock Vina software package (http://vina.scripps.edu/index.html)^46^. The PDB file 4A7U of the SOD1 dimer^36^ was downloaded from http://www.rcsb.org/pdb/home. The molecular model of NBD compounds was created in pdb format with ChemBioDraw Ultra 12.0 software (http://software.informer.com/getfree-chembio3d-ultra-12.0/). Free energy minimization of compounds was performed with the MM2 Program of ChemBioDraw Ultra 12.0 software. Putative binding sites formed between small compounds and SOD1 were checked with AutoDock Tools 1.5.6rc3. The highest-scoring nine conformations were predicted for each interaction by the Vina scoring function.

### Cell viability test

Cell lines of breast cancer MDA MB468, and transformed human keratinocytes NCTC 2544 were seeded at 2 × 10^4^ cells per well in 96-well microtitre plates. Each NBD compound was tested by standard serial dilutions in cultures incubated for 72 h at 37 °C by a colorimetric assay based on the conversion of tetrazolium dye to blue formazan by live mitochondria^47^. Eight repeats were performed for each concentration of the compound to be tested. The IC_50_ value of NBD compounds was determined by measuring colour intensity at 570 nm.

### Statistical analysis

Differences between the control and the treated cells were assessed with the paired Student’s t-test, and p < 0.05 was considered statistically significant. Data analysis was performed with SigmaPlot v10.

## Additional Information

**How to cite this article**: Sakanyan, V. *et al.* Activation of EGFR by small compounds through coupling the generation of hydrogen peroxide to stable dimerization of Cu/Zn SOD1. *Sci. Rep.* **6**, 21088; doi: 10.1038/srep21088 (2016).

## Supplementary Material

Supplementary Information
