# Supplementary material for: Activation of EGFR by small compounds through coupling the generation of hydrogen peroxide to stable dimerization of Cu/Zn SOD1
Source: Sci Rep. 2016 Feb 17;6:21088. doi: 10.1038/srep21088 (PMC4756678; doi:10.1038/srep21088)
Supplement: Supplementary Information [file srep21088-s1.pdf]

Supplementary Information

Activation of EGFR by small compounds through coupling the generation of hydrogen peroxide to stable dimerization of Cu/Zn SOD1

Vehary Sakanyan[1, 2,*], Philippe Hulin[3], Rodolphe Alves de Sousa[4], Viviane A. O. Silva[5], Artur Hambardzumyan[6], Steven Nedellec[3], Christophe Tomasoni[1], Cédric Logé[1], Charles Pineau[7], Christos Roussakis[1], Fabrice Fleury[5], Isabelle Artaud[4]

Supplementary Text

Synthesis of NBD compounds. In a first step, reaction of NAC methyl ester under its sodium thiolate form with NBD-Cl led to CN 009543V. This reaction was performed very quickly to avoid the secondary addition of NAC methyl ester thiolate on CN 009543V leading to the formation of the corresponding disulphide. In a second step, CN 009543V was oxidized either with dioxirane (DMD) or 3-chloroperbenzoic acid (mCPBA). This oxidation did not result in the selective formation of the sulphoxide relative to the sulphone, since oxidation of sulphoxide was faster than that of the starting thioether. Thus, it required an excess of oxidant to consume the entire starting product. However, the same sulphoxide/sulphone (3/7) ratio was always obtained with 3 to 5 equiv. of oxidant. These compounds, referred to as CN 009616V and CN 009617, were easily separated by flash chromatography on silica gel and characterized by high-resolution mass spectrometry (HRMS), ^{1}H and ^{13}C NMR. All solvents and chemicals were purchased from SDS and Aldrich. ^{1}H NMR and ^{13}C NMR spectra were recorded on a Bruker Avance-500 spectrometer and chemical shifts were reported in ppm downfield from TMS. Electrospray ionization (ESI) mass spectrometry analyses were obtained using a Thermo Fisher Exactive (Orbitrap). UV spectroscopy and fluorescence spectroscopy measurements were recorded on a Shimadzu UV-2700 spectrophotometer and a Hitachi F-7000 FL spectrophotometer, respectively.

Synthesis of methyl *N*-acetyl-*S*-(7-nitro-2,1,3-benzoxadiazol-4-yl)cysteinate (CN 009543V). Powdered NaH (40.5 mg, 1.69 mmol) was added to a tetrahydrofuran solution of N-acetyl cysteine methyl ester (300 mg, 1.69 mmol in 20 mL). The solution darkened after addition at 0°C of powdered NBD-Cl (337 mg, 1.69 mmol) and was then immediately evaporated to dryness. The residue was dissolved in CH_2Cl_2 and, after eliminating NaCl by filtration, the compound was purified by column chromatography (SiO_2, CH_2Cl_2/Acetone, 90/10) to yield 150 mg of yellow powder. Yield: 26%. ^{1}H NMR (500 MHz, $CDCl_3$) δ (ppm): 8.45 (d, J = 7.8 Hz, 1H), 7.5 (d, J = 7.8 Hz, 1H), 6.38 (d, J = 5.9 Hz, 1H), 5 (q, J = 5.9 Hz, 1H), 3.86 (dd, J = 13.8 Hz, 6.3 Hz, 1H), 3.82 (s, 3H), 3.77 (dd, J = 13.8 Hz, 4.7 Hz, 1H), 2.05 (s, 3H); ^{13}C NMR (500 MHz, $CDCl_3$) δ (ppm) 23.1, 33.4, 51.6, 53.3, 122.2, 130.5,

133.5, 139.1, 142.5, 149.3, 170.1, 170.3; UV/vis in PBS: λmax 418.5 nm; HRMS(ESI$^-$) (m/z): $[M\text{-}H]^-$ calcd. for $C_{12}H_{12}N_4O_6S$, 339.0405; found, 339.0417.

Synthesis of methyl *N*-acetyl-3-[(7-nitro-2,1,3-benzoxadiazol-4-yl)sulphinyl]alaninate (CN 009616V) and methyl *N*-acetyl-3-[(7-nitro-2,1,3-benzoxadiazol-4-yl)sulphonyl]alaninate (CN 009617V). CN 009543V (90 mg, 0.26 mmol) was diluted in dichloromethane (25 mL), cooled to 0°C and mCPBA (137 mg, 0.79 mmol) in dichloromethane (10 mL) was added slowly. Stirring was maintained for 1 h then saturated aqueous NaCl (50 mL) was added. The organic phase was extracted twice, dried over Na_2SO_4 and concentrated. The yellow powder was purified by column chromatography (SiO_2, CH_2Cl_2/Acetone, 80/20) to afford 46 mg of sulphone (CN 009617V) as a white solid (Yield: 47%) and 20 mg of sulphoxide (diastereoisomers mixture 60/40) as a pale yellow solid (Yield: 21%).

CN 009616V: The diastereoisomers were not separated; however, the allocation 1H and 13C was completely performed.

Diastereoisomer 1: ^{1}H NMR (500 MHz, acetone D6) δ (ppm): 8.87 (d, *J* = 7.5 Hz, 0.6H); 8.25 (d, *J* = 7.5 Hz, 0.6H); 8 (d, *J* = 10 Hz, 0.6H); 4.97 (td, *J* = 10 Hz, 3.7 Hz, 0.6H); 3.87 (dd, *J* = 13.6 Hz, 10 Hz, 0.6H), 3.64 (s, 1.8H); 3.58 (dd, *J* = 13.6 Hz, 3.7 Hz, 0.6H); 1.29 (s, 1.8H); ^{13}C NMR (500 MHz, acetone D6) δ (ppm) 22.3, 48.5, 53.1, 57.2, 129.5, 129.7, 131.4, 143.4, 144.3, 147.4, 169.9, 171.4.

Diastereoisomer 2: ^{1}H NMR (500 MHz, acetone D6) δ (ppm): 8.86 (d, *J* = 7.5 Hz, 1H); 8.16 (d, *J* = 7.5 Hz, 1H); 7.44 (d, *J* = 7.9 Hz, 1H); 5.06 (td, *J* = 7.9 Hz, 4.1 Hz, 1H); 4.13 (dd, *J* = 14.3 Hz, 4.1 Hz, 1H); 3.7 (s, 3H); 3.63 (dd, *J* = 14.3 Hz, 7.9 Hz, 1H); 1.56 (s, 3H). ^{13}C NMR (500 MHz, acetone D6) δ (ppm) 22.3, 46.2, 53.1, 54.9, 129.5, 129.7, 131.4, 143.4, 144.3, 147.4, 169.9, 171.4.

UV/vis in PBS: λmax 349.5 nm; HRMS (ESI$^+$) (m/z): $[M+H]^+$ calcd. for $C_{12}H_{12}N_4O_7S$, 357.0499; found: 357.0491.

CN 009617V: ^{1}H NMR (500 MHz, acetone D6) δ(ppm): 8.89(d, *J* = 7.5 Hz, 1H), 8.45 (d, *J* = 7.5 Hz, 1H), 7.62 (d, *J* = 8.4 Hz, 1H), 4.93(td, *J* = 8.4 Hz, 3.9 Hz, 1H), 4.27 (dd, *J* = 15.3 Hz, 3.9 Hz, 1H), 4.18 (dd, *J* = 15.3 Hz, 8.4 Hz, 1H), 3.66 (s, 3H), 1.65 (s, 3H); ^{13}C NMR (500 MHz, acetone D6) δ(ppm) 22.3, 48.9, 53.2, 56.4, 131, 135, 135.8, 141.2, 144.7, 147.6, 170.2, 170.4; UV/vis in PBS: λmax 319 nm; HRMS(ESI$^+$) (m/z): $[M+H]^+$ calcd. for $C_{12}H_{13}N_4O_8S$, 373.0449; found, 373.0440.

NMR Data

^{1}H and ^{13}C NMR for **CN 009617V** in acetone D6:

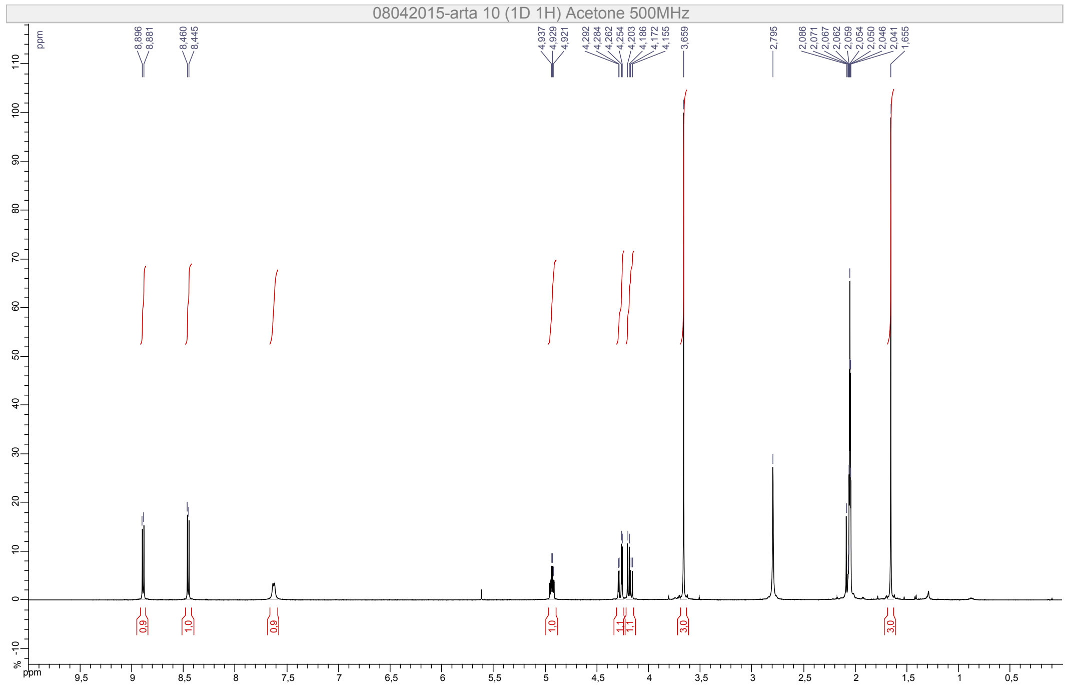

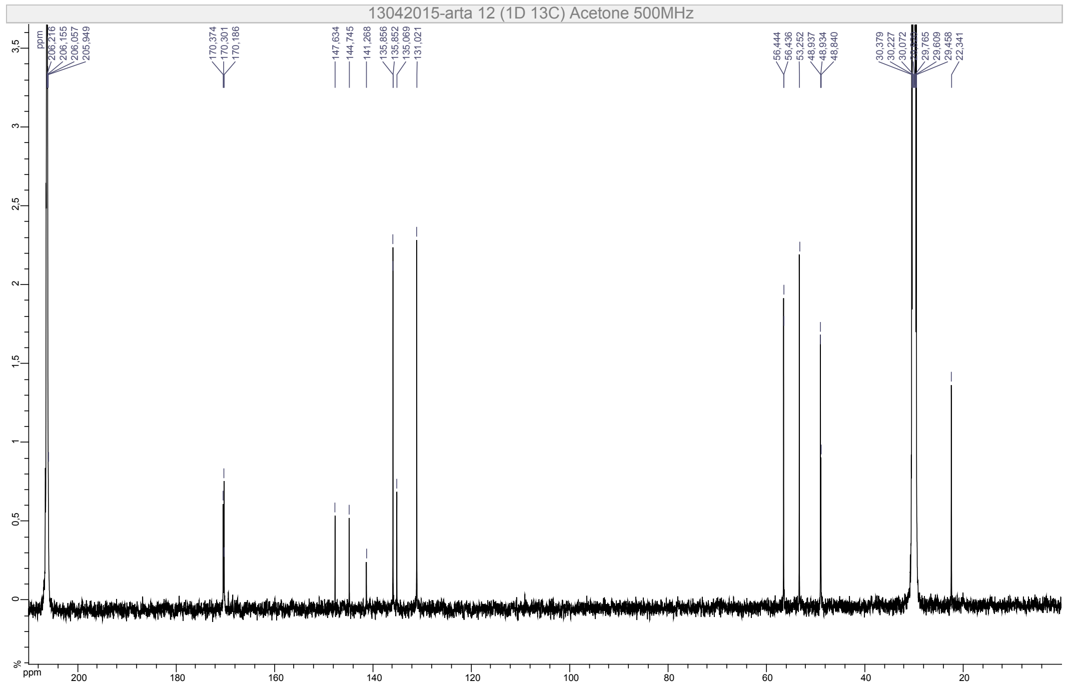

^{1}H and ^{13}C NMR for **CN 009616V** in acetone D6:

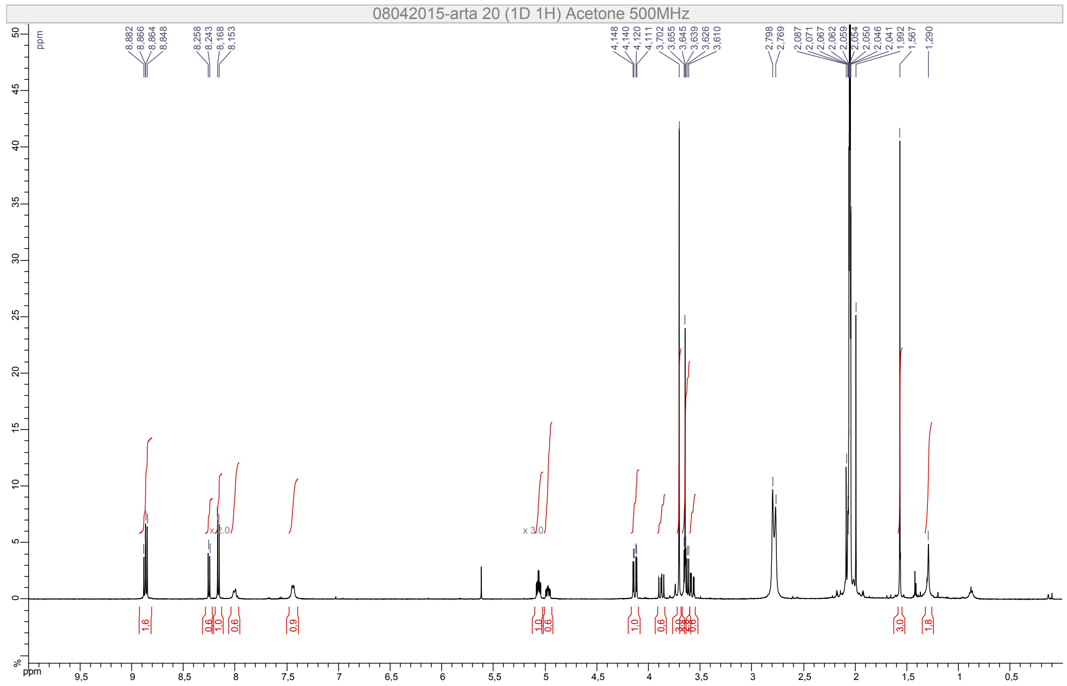

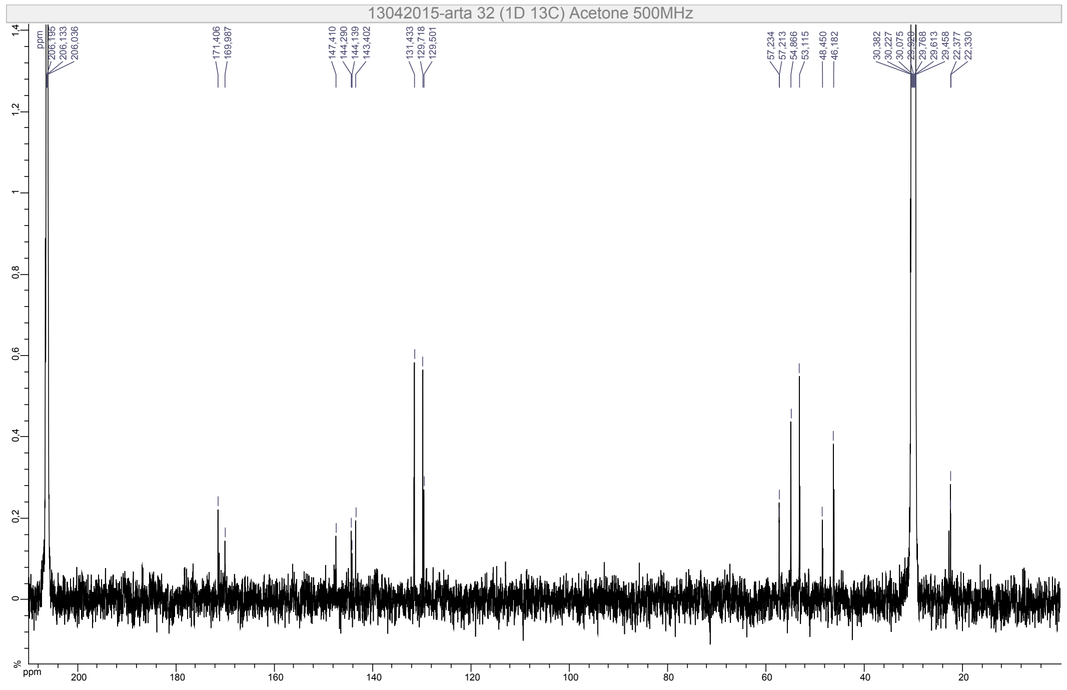

^{1}H and ^{13}C NMR for **CN 009543V** in $CDCl_3$:

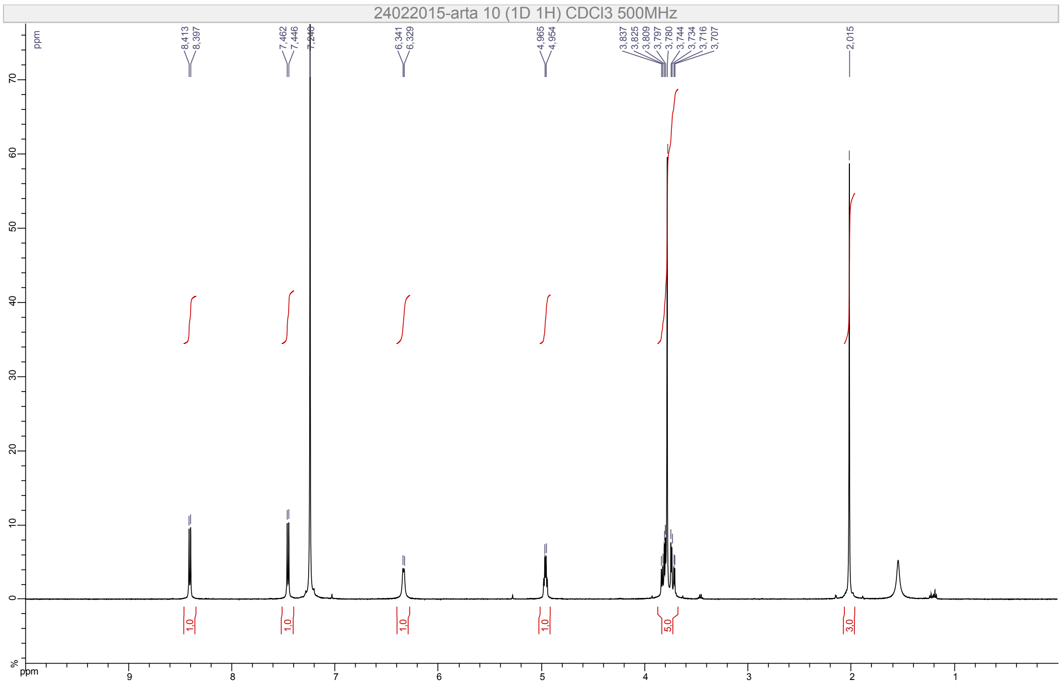

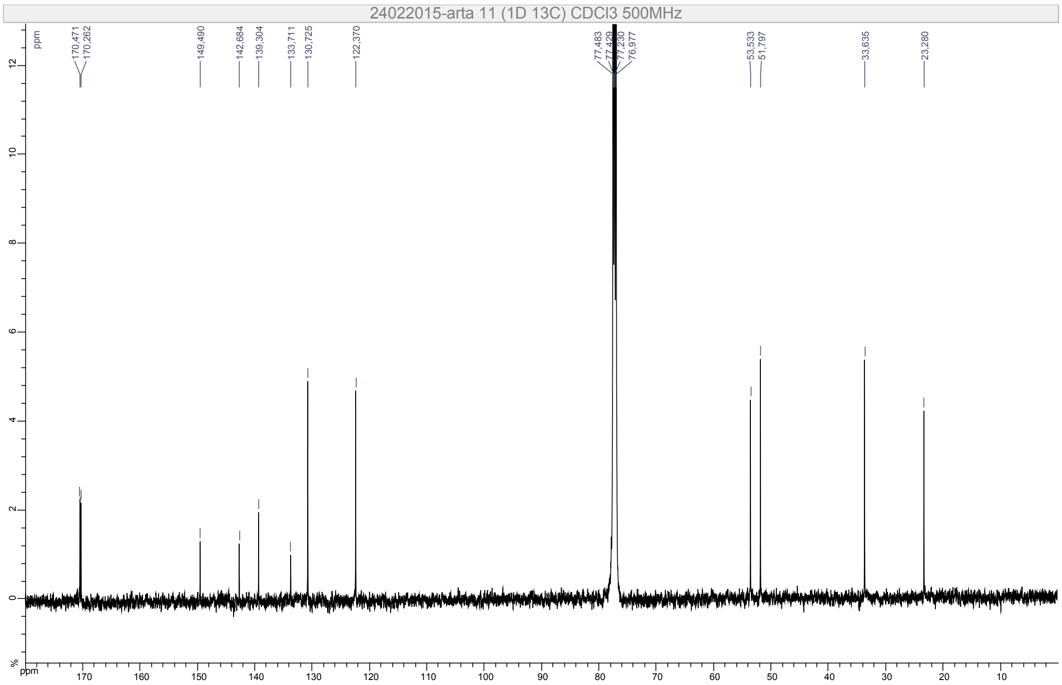

Supplementary Figures & Tables

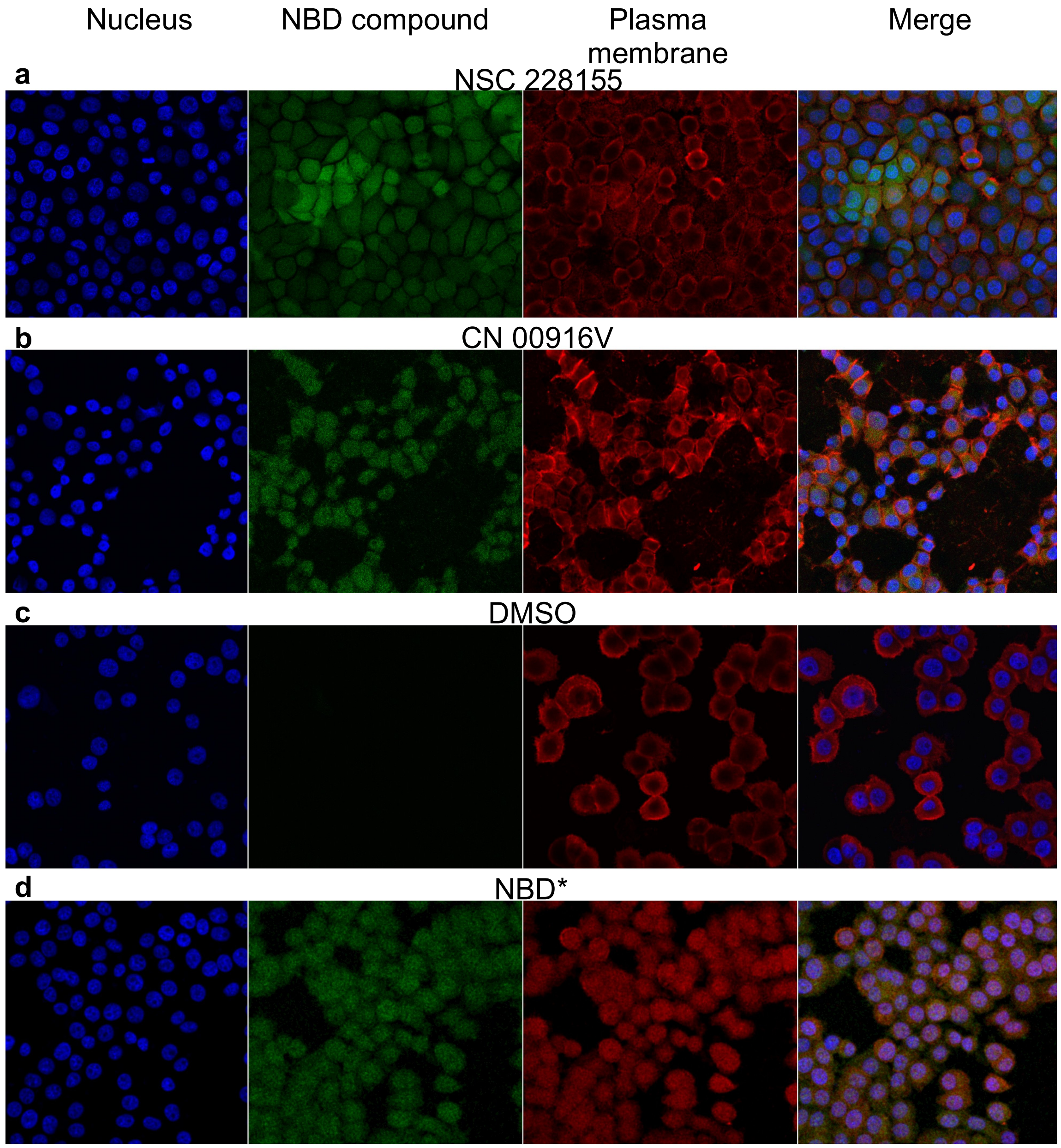

Fig. S1. Fluorescent detection of NBD compounds in cells.

Multicolour presentation of the nucleus (Hoechst), NBD compounds and plasma membrane-located EGFR (recognized with biotinylated anti-EGFR and neutravidin-conjugated DyLight 649) in MDA MB468 cells.

Low fluorescence emitted from cells exposed to CN 009616V (**b**), DMSO (**c**), and NBD* (**d**) was recorded at higher sensitivity compared to cells exposed to NSC 228155 (**a**).

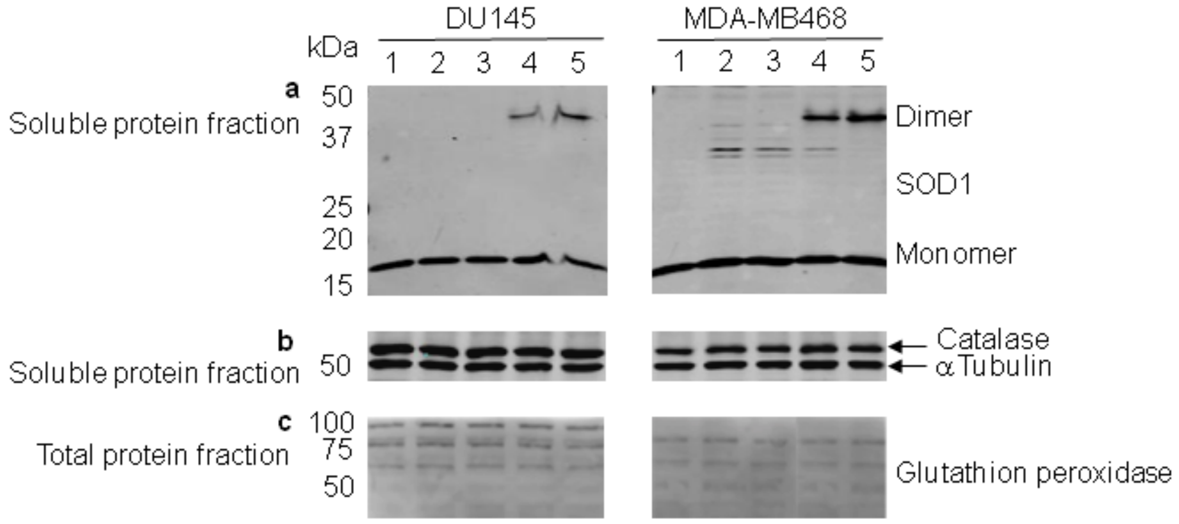

Fig. S2. Relative level and oligomerization of enzymes SOD1, catalase and glutathione peroxidase in cancer cells exposed to compound NSC 228155.

Prostate cancer DU145 and breast cancer MDA MB468 cells were incubated with 1 - vehicle (DMSO) for 10 min; 2 and 3 - EGF (150 ng/ml) for 5 and 10 min, respectively; 4 and 5 - NSC 228155 (100 μM) for 5 and 10 min, respectively. Soluble protein fractions (supernatant) were used for detection of SOD1 and catalase. No glutathione peroxidase was detected in the soluble fraction of cells whereas multiple bands were detected in the total protein fraction (lysed cell extracts without centrifugation) using anti-glutathione ½ antibody. α-Tubulin was used as a loading control.

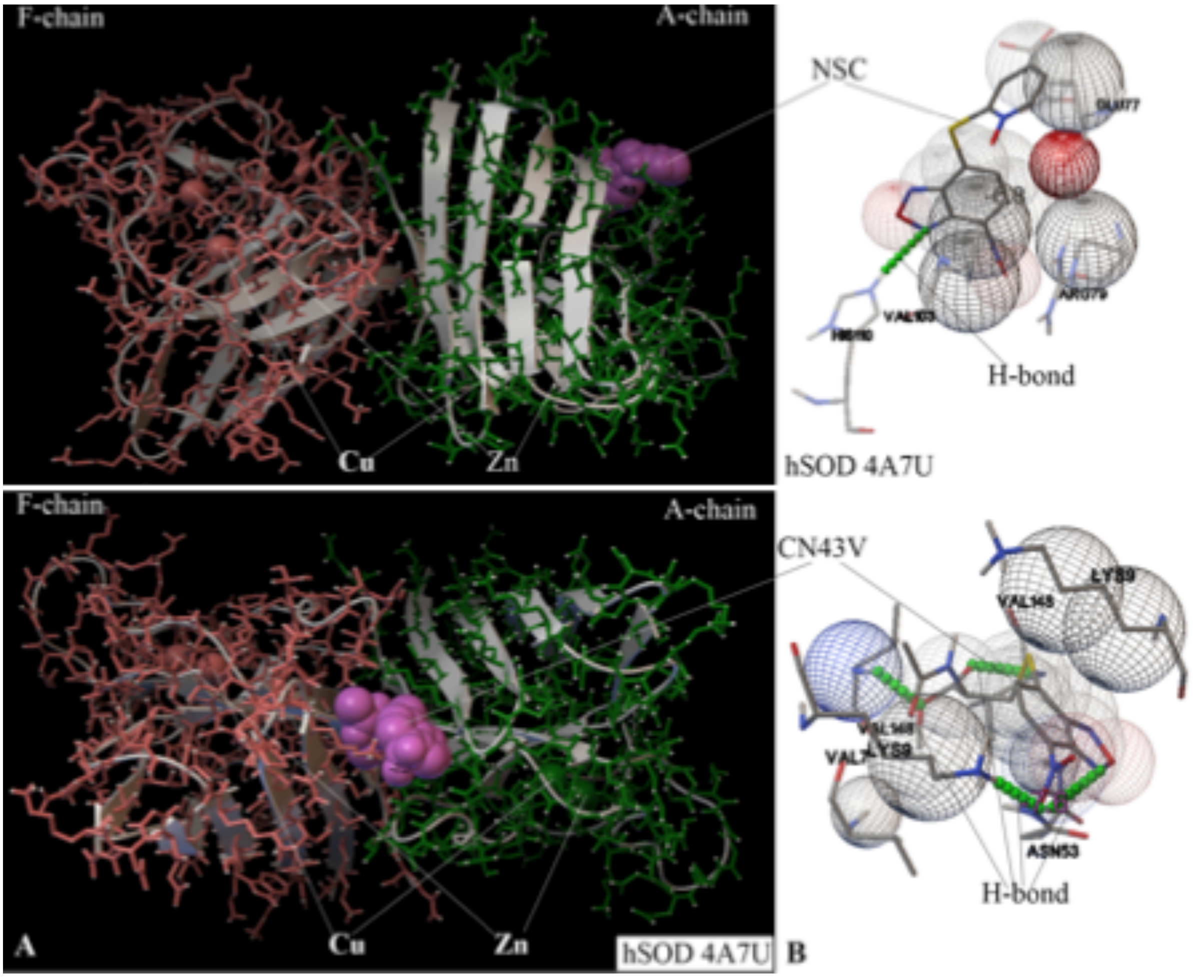

Fig. S3. Docking of NSC 228155 (NSC) and CN 009543 (CN43V) compounds in 4A7U, the dimer of human Cu/Zn SOD1.

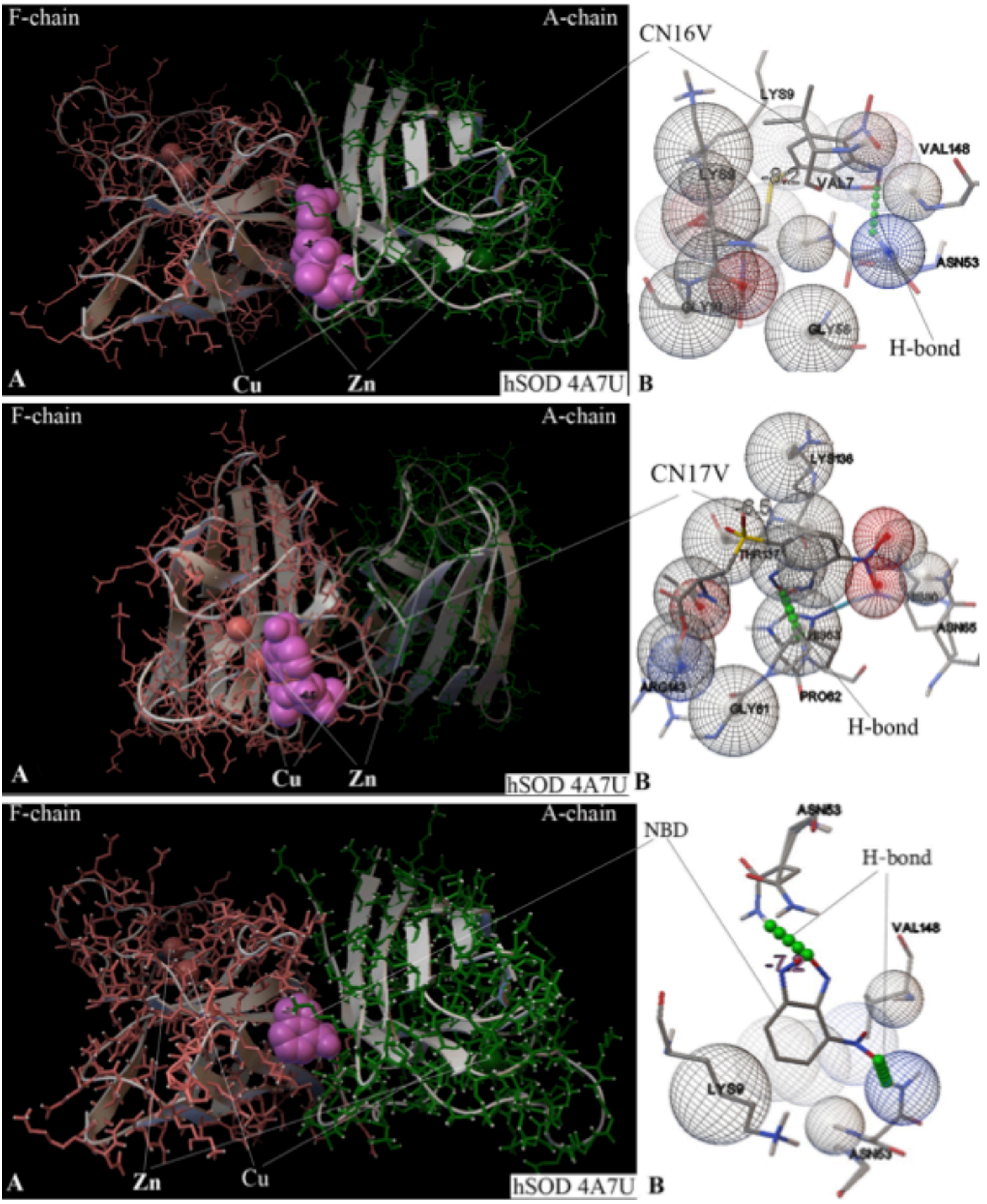

Fig. S4. Docking of CN 00916V (CN16V), CN 00917V (CN17V) and NBD* (NBD) compounds in 4A7U, the dimer of human Cu/Zn SOD1.

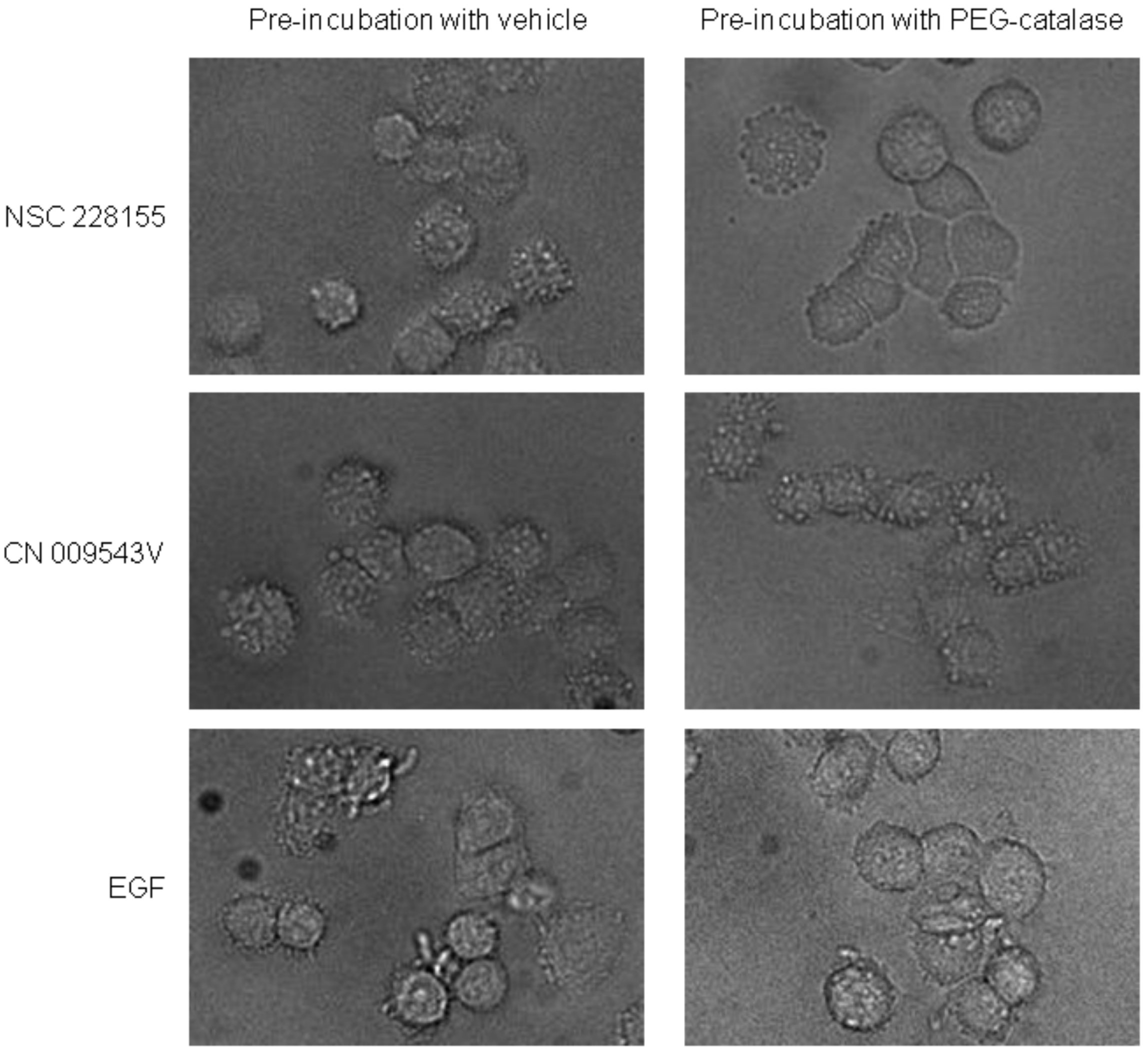

Fig. S5. Microscopic detection of blebbing in cancer cells exposed to NBD compounds.

MDA MB468 cells were pre-incubated DMSO or with PEG-catalase (500 U/ml) and then incubated with NSC 228155 (100 μM), CN 009543V (100 μM) or EGF (500 ng/ml) for 12 min.

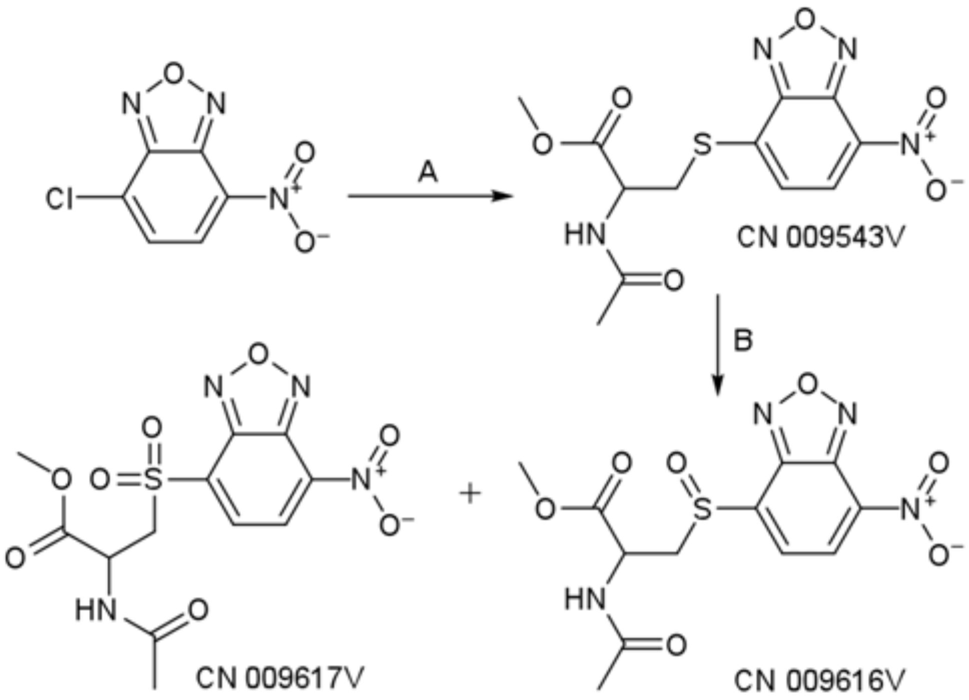

Fig. S6. Synthetic route for NBD compounds.

A, N-acetyl-L-cysteine methyl ester, NaH, THF, 0°C, 5 min. **B**, mCPBA, CH_2Cl_2, 0°C, 2 h.

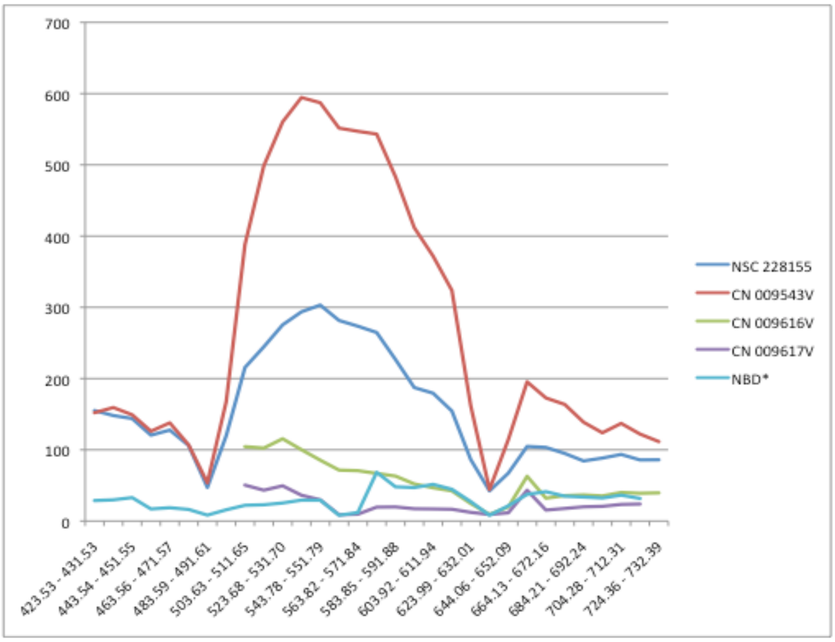

Fig. S7. Fluorescence recorded in the supernatant fraction of cell lysates obtained after exposure to NBD compounds.

Table S1. Interaction of NBD compounds with the dimer structure of human Cu/Zn SOD1

The docking analysis of the best conformation is shown for each NBD compound bound to 4A7U. A and F are the chains of SOD1. Hydrogen bond is shown in red.

**CN 009616V**	**CN 009617V**	**CN 009543**	**NSC 228155**	**NBD***
-8.2 kkal/mol	-6.5 kkal/mol	-7.6 kkal/mol	-6.8 kkal/mol	-7.2 kkal/mol
A:Val7				
		F:Val7		
A:Lys9				
F:Lys9		F:Lys9(H)		F:Lys9
A:Gly10				
A:Asn53 (H)		A:Asn53(H)		A:Asn53(H)
				F:Asn53(H)
A:Gly56				
	F:Gly61			
	F:Pro62			
	F:His63 (H)			
			A:Glu77	
			A:Arg79	
	F:His80			
			A:Val103	
			A:His110(H)	
	F:Lys136			
	F:Thr137			
	F:Arg143			
A:Val148		A:Val148(H)		A:Val148
		F:Val148(H)		

Table S2. Toxicity of NBD compounds (IC_{50}) in cell lines MDA MB468 and NCTC 2544. The viability of cells was determined after incubation with NBD compounds for 48 h.

Compound	MDA MB468	NCTC 2544
NBD*	2.67 μM	10.91 μM
NSC 228155	0.15 μM	2.71 μM
CN 009543V	0.71 μM	4.41 μM
CN 009616V	nd (>10 μM)	nd (>30 μM)
CN 009617V	nd (>10 μM)	nd (>30 μM)
